# Scorpion-Derived Antiviral Peptides with a Special Focus on Medically Important Viruses: An Update

**DOI:** 10.1155/2021/9998420

**Published:** 2021-09-04

**Authors:** Moulay Abdelmonaim El Hidan, Mehdi Ait Laaradia, Omar El Hiba, Ahmed Draoui, Abdelmohcine Aimrane, Kholoud Kahime

**Affiliations:** ^1^Laboratory of Biotechnology and Valorization of Natural Resources, Faculty of Applied Sciences, Ibn Zohr University, Agadir, Morocco; ^2^Faculty of Sciences Semlalia, Cadi Ayyad University, Marrakech, Morocco; ^3^Nutritional Physiopathology Team, Faculty of Sciences, Chouaib Doukkali University, El Jadida 24000, Morocco; ^4^Laboratory of Clinical and Experimental Neurosciences and Environment, Faculty of Medicine and Pharmacy, Cadi Ayyad University, Morocco; ^5^Metabolic Platform, Biochemistry Laboratory, Faculty of Medicine, Cadi Ayad University, Marrakech, Morocco; ^6^SAEDD Laboratory, School of Technology Essaouira, Cadi Ayyad University of Marrakesh, Morocco

## Abstract

The global burden of viral infection, especially the current pandemics of SARS-CoV-2, HIV/AIDS, and hepatitis, is a very risky one. Additionally, HCV expresses the necessity for antiviral therapeutic elements. Venoms are known to contain an array of bioactive peptides that are commonly used in the treatment of various medical issues. Several peptides isolated from scorpion venom have recently been proven to possess an antiviral activity against several viral families. The aim of this review is to provide an up-to-date overview of scorpion antiviral peptides and to discuss their modes of action and potential biomedical application against different viruses.

## 1. Introduction

Viruses are obligatory intracellular parasites that infect and invade host cells to replicate. Throughout history, humans have been exposed to a multitude of viral infections and diseases. The existence of some viruses has been reported since humans first appeared on earth, while others have been contracted from animals. With the growing human population, wildlife is increasingly affected and human-induced habitat changes are forcing greater wildlife-human interactions, with the consequence being that there are more and more zoonotic viruses provoking epidemics of serious human diseases. Nevertheless, new viruses are not the only ones that affect humans; many long-known viruses continue to cause widespread problems.

Indeed, the SARS-CoV-2 epidemic was initially thought to have started via zoonotic transmission associated with the seafood market in China (Wuhan). Human-to-human transmission was later recognized as playing a significant role in the subsequent epidemic. The disease resulting from this virus was named coronavirus 19 disease, and a pandemic was declared by the World Health Organization [1].

In fact, although considerable progress has been made, it has not yet been fully possible to develop an effective vaccine for some of the most infectious viruses, such as HIV [2]. Even when vaccines exist, problems may continue especially with RNA viruses, because of their high genetic variation rates and mutation-occurring frequency, which are expressed by their high ability to adapt and exploit various conditions [3]. Moreover, decreased sensitivity to actual drugs such as with herpes and hepatitis viruses constitute a challenge to conventional antimicrobial therapies [2, 4]. Subsequently, additional antiviral agents need to be developed to overcome problems of emerging and reemerging infectious viral diseases.

Several antimicrobial peptides isolated from scorpion venoms have shown great potential as antiviral agents [5]. Applications of modern techniques such as proteomic and transcriptomic analyses showed that the scorpion peptides are considerably more complex than previously realized. Actually, single scorpion venom commonly comprises up to several hundred diverse components [6, 7], and together with assessments of scorpion diversity, this has provided estimates of nearly 100,000 biologically active peptides in scorpion venoms [8]. Hence, scorpion venom is a complex mixture of peptides and proteins with a wide range of biological activities, providing an unexploited resource for application in the design and development of peptide drugs [9, 10].

The main goal of this review is to provide an overview on antiviral peptides discovered or developed from scorpion venom.

## 2. Scorpion Venom Composition

As evidenced by many authors, scorpion venoms are a complex mixture of organic and inorganic compounds. While not as well studied, the nontoxic fraction of scorpion venom has been characterized. In general, scorpion venoms were found to contain sodium, potassium, calcium, as well as trace amounts of magnesium, phosphorus, iron, silicon, boron, zinc, and copper [11], as well as, enzymes (e.g., hyaluronidase and phospholipase), enzyme inhibitors and bioamines (including serotonin and histamine). While carbohydrates and lipids have been detected in scorpion venom (such as mucopolysaccharides and phospholipids) [12].

Unlike the nontoxic components, toxic fractions in scorpion venom have been extensively studied and intensive research into their structure and function continues. In general, scorpion polypeptides are divided into two groups: the disulfide-bridged peptides (DBPs) and the nondisulfide-bridged peptides (NDBPs) [13, 14]. They consist of 13-50 amino acid residues [13, 14], and NDBPs are classified into two classes: cationic and highly acidic peptides. The acidic peptides possess generally coiled-coil structures, and their activities have not yet been identified [14, 15]. The cationic peptides from scorpions are usually *α*-helical and amphipathic molecules that may have either antimicrobial or bradykinin-potentiating activities or act as signalling molecules involved in the modulation of immune responses. Most cationic peptides present an array-spectrum and nonspecific activity against a varied range of microorganisms, including virus, Gram-positive and Gram-negative bacteria, protozoa, and fungi [15]. The other components of the toxic fraction of scorpion venom are toxins that act specifically on Na^+^, K^+^, Cl^−^, or Ca^2+^ ion channels. Generally, they are composed of 23-98 amino acid residues cross-linked by 3-5 disulfide bridges [16].

## 3. AVP Discovery

The first antiviral peptide developed from scorpion venom was the recombinantly expressed scorpine (Rscp). Rscp was derived in 2008 from scorpine, a 75-amino acid peptide purified from *Pandinus imperator* venom, and had shown a great ability to inhibit virus dengue-2 replication in C6/36 mosquito cell [17]. However, the Hp1090 was the first reported natural antiviral peptide, discovered through cDNA technology from the gland of *Hererometrus peterssi* in 2011. The Hp1090 is an amphipathic alpha-helical peptide, with viricidal activities against hepatitis C virus (IC_50_ of 7.62 *μ*g/ml) [18]. Later, in the same year, antiviral activity of the optimized mucroporin-M1 was demonstrated against measles virus, the SARS coronavirus, and influenza A virus subtype H5N1. Fourteen antiviral peptides are discovered or derived from scorpion venom natural peptides [10, 19, 20].

## 4. Main Scorpion AVPs and Targeted Virus

**Figure 1:**
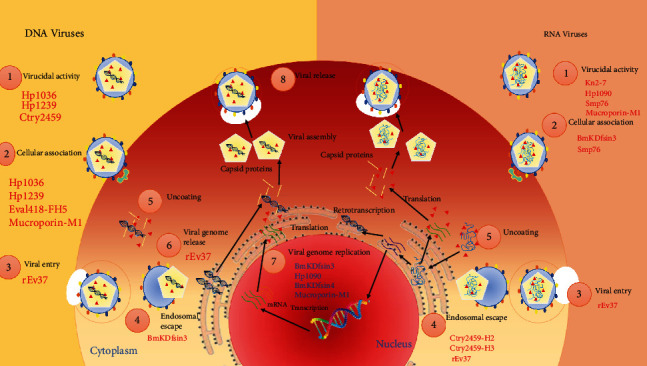
The possible inhibition sites targeted by various scorpion antiviral peptides.

Viruses are commonly described as subcellular, infectious agents. They are obligate intracellular parasites that infect and take possession of a targeted cell in order to replicate. Viruses display a wide diversity of shapes and sizes; currently, more than 6000 virus species have been fully described of the millions of types of viruses. According to the mechanism of mRNA production (Baltimore classification), seven groups can be recognized: double-stranded (ds) DNA viruses, single-stranded (ss) DNA viruses, dsRNA viruses, (+)ss RNA viruses, (-)ss RNA viruses, ssRNA reverse transcriptase (RT) viruses, and dsDNA-RT viruses [21]. All AVPs originated from scorpion source are summarized in Table 1 and Figure 1 with their mode of action.

### 4.1. Antihuman Immunodeficiency Viruses-1 (HIV-1) Peptide

The human immunodeficiency viruses (HIV) are an ssRNA-RT, belonging to the *lentivirus* genus; part of the family of Retroviridae. Over time, they induce acquired immunodeficiency syndrome (AIDS). They are transmitted as single-stranded, positive sense, enveloped RNA viruses. After cell targeting, viral RNA genome is reverse transcribed into double-stranded DNA by a virally reverse transcriptase. Two types of HIV have been identified; HIV-1 and HIV-2. HIV-1 is the first discovered and the most virulent and infective in comparison with HIV-2 and is the cause of the majority of HIV in the world [22].

Kn2-7 an anti-HIV-1 peptide consists of an alpha-helical peptide of 13-amino acid peptide, developed from a native *Mesobuthus martensii* scorpion peptide (BmKn2) by replacing glycine, alanine, and serine with lysine or arginine (G3K, A4R, and S10R) in order to increase the net positive charge [22]. Kn2-7 had a potent anti-HIV activity. In fact, it might exert its antiviral activity on all of 13 strains of a standard reference panel of HIV-1 subtype B pseudotyped virus (PV) with CCR5-tropic and CXCR4-tropic NL4-3PV. Moreover, it has an EC_50_ of 2.76 *μ*g/ml (1.65 *μ*M), with a low cytotoxicity to host cells with a selective index (SI) of 13.93 [22]. Kn2-7 may inhibit HIV-1 by damaging virus particle itself, especially if we know that viral infectivity was inhibited only when Kn2-7 had chance to interact directly with the virus particle. Authors suggested that Kn2-7 aggregates and inserts into viral envelope so that the hydrophobic peptide region aligns with the lipid core region and the hydrophilic peptide regions form the interior region of the pore, with the help of positive charge of peptide somehow [22].

### 4.2. Antihepatitic C Virus (HCV) Peptides

HCV is a (+)ssRNAvirus and a member of *Hepacivirus* genus, part of the family Flaviviridae. It consists of a small (55 to 65 nm in diameter) lipid membrane, which envelopes a positive-sense single-stranded RNA. HCV is characterized by two viral envelope glycoproteins; E1 and E2 embedded in the lipid envelope. HCV causes hepatitis C and some cancers such as liver cancer and lymphomas in humans [23].

#### 4.2.1. BmKDfsin3 Peptide

BmKDfsin3 is a scorpion defensing peptide developed from *M. martenssi Karsch*. It consists of 38 amino acid residues, with six cysteine residues forming three pairs of disulfide bonds [24]. The IC_50_ is around 3.35 *μ*M. BmKDfsin3 can inhibit HCV replication and affect the attachment and postentry stages of the viral infection cycle at noncytotoxic concentrations with low cytotoxicity (IC_50_ around 60.63 *μ*M to Huh7.5.1 cells). Otherwise, HCV replication inhibition by BmKDfsin3 is related to the suppression of the classical p38 MAPK signal pathway [24].

#### 4.2.2. Hp1090 Peptide

Hp1090 is the first natural AVP characterized from cDNA library from the venom gland of the scorpion *Heterometrus petersii*. Hp1090 is an amphipathic-helical peptide with high inhibition activity of HCV (IC_50_ of 5 *μ*M) at noncytotoxic concentration. This AVP has the ability to prevent the initiation step of HCV infection by direct interaction with HCV virus and readily disrupting their phospholipid membrane. Moreover, Hp1090 inhibited the amplification of viral particle RNA in Huh7.5.1 cell with a greater ability than INF-*α* [18].

#### 4.2.3. rEv37 Peptide

The rEv37 is an AVP derived from Ev37, which is 78 amino acid residues purified from *Euscopio psvalidus* venom. rEv37 is a scorpine-like peptide, including a CS*α*/*β* motif and has six cysteines that can form three pairs of disulfide bridges. rEv37 had little effect on the viral replication of HCV, and it is also slightly virucidal for the virus particles but not for the HCV genome. Thus, rEv37 inhibited viral entry to the host cells by affecting internalization (fusion) stage of the HCV life cycles, via low pH-dependent endocytosis pathway [25].

#### 4.2.4. Smp76 Peptide

The Smp76 is a natural peptide purified from *Scorpio maurus palmatus* venom. It consists of 76 amino acids with six cysteine residues, stabilized by three disulfide bridges. Smp76 presents an anti-HCV activity with an approximate IC_50_ of 0.01 *μ*g/ml and no toxic or hemolytic effects *in vitro* at a concentration 1000-fold higher than that required antiviral activity. Smp76 ability to prevent the early stages of HCV life cycle is most probably related to its interaction with viral particles. The authors had suggested that HCV could be neutralized by targeting its envelope or host factors related to the mature viral particle [23].

#### 4.2.5. Ctry2459 and Its Derived Peptides for Anti-HCV Defense

The Ctry2459 is a peptide discovered from a cDNA library from the scorpion *Chaerilus tryznai.* It consists of 9 amino acid residues with helical and amphipathic structure. With an IC_50_ of 1.84 *μ*g/ml and without cytotoxic effect on Huh7.5.1 cells (CC_50_ around 79.8 *μ*g/ml), Ctry2459 exhibits a striking inhibitory ability against HCV infection by directly destabilizing the viral structural integrity and thus decreasing the initiation of HCV infection [26].

Nevertheless, this antiviral peptide was unable to inhibit acquired infection, due to its poor cellular uptake and aggregation in cytoplasmic endosomes. In order to improve cellular uptake and intracellular distribution, two new histidine-rich peptides (Ctry2459-H2 and Ctry2459-H3) were designed based on the native Ctry2459. The two new peptides had the same *α*-helix structure as the wild-type Ctry2459, but enhance amphipathic *α*-helix conformation. Ctry2459-H2 and Ctry2459-H3 had higher bioavailability and efficiently enter the cells, break through the endosomes, interact with the mature viral particles, and significantly inhibit the established HCV infection at cellular level, though presenting even inferior cytotoxic and hemolytic activities compared to the native peptide [27].

### 4.3. Antihepatitic B Virus (HBV) Peptides

HBV is partially double-stranded DNA virus [28], a species of the genus *Orthohepadnavirus*, and belongs to the Hepadnaviridae family. It consists of an outer lipid envelope and an icosahedral nucleocapsid core composed of protein that encloses the viral DNA and a DNA polymerase with a reverse transcriptase activity [29]. HBV is responsible of hepatitis disease [30], cirrhosis, and hepatocellular carcinoma [31, 32].

#### 4.3.1. BmKDfsin4 Peptide

The BmKDfsin4 consists of a small cationic peptide with six cysteine residues, and BmKDfsin4 is a scorpion-derived defensin with antibacterial and Kv1.3-blocking activities. Additionally, BmKDfsin4 had been shown to exhibit powerful inhibitory activity against HBV replication by reducing the production of HBeAg (IC_50_ = 3.95 *μ*M), HBsAg (IC_50_ = 2.28 *μ*M), and HBV DNA (IC_50_ = 1.26 *μ*M) in cell culture medium and the production of intracellular HBsAg, HBV core protein, HBx protein, and HBV RT with extremely low cytotoxic and hemolytic effects [33].

#### 4.3.2. Mucroporin-M1 Peptide

The mucroporin-M1 is an antiviral peptide developed from mucroporin, a cationic host peptide with 17-amino acid cloned from the venomous glands of the scorpion *Lychas mucronatus* [20].

Mucroporin optimization was carried out by substituting glycine and proline residues with lysine or arginine (G3R, P6K, G10K, and G11R) in order to improve the net positive charge of the hydrophilic side. Mucroporin-M1 had shown a highly suppressive ability against HBV both in vitro and in vivo. Thus, mucroporin-M1 reduces the amount of extracellular HBsAg, HBeAg, and HBV DNA productions of HepG2.2.15 cells in vitro. Furthermore, this peptide inhibits HBV capsid DNA and HBV intracellular RNA replication intermediates and the HBVcore protein in the cytoplasm of HepG2.2.15 cells. In vivo, mucroporin-M1 inhibits significantly HBV replication via reduction in HBV promoter activity. In fact, mucroporin-M1 activates the mitogen-activated protein kinases (MAPKs) that lead to decrease in the binding of HNF4*α* to the precore/core promoter region and reduce the expression of HNF4*α* [34].

The IC_50_ values of mucroporin-M1 against HBsAg and HBeAg production were 20.6 and 4.9 *μ*M, respectively. While the production of HBV progeny DNA was also inhibited in a dose-dependent manner by mucroporin-M1, with an IC50 of 11 *μ*M [34].

### 4.4. Antiherpes Simplex Virus-1 (HSV-1) Peptides

HSV-1 belongs to the *Simplex virus*, one of the human Herpesviridae families. It consists of a relatively large, double-stranded, linear DNA genome encased within an icosahedral protein cage called capsid, which is wrapped in a lipid bilayer called envelope. HSV-1 induces herpes labialis, commonly known as cold sores [35].

#### 4.4.1. Hp1036 and Hp 1239 Peptides

Hp1036 and Hp 1239 are cationic and amphipathic scorpion venom peptides cloned from *Heterometus petersii* with an *α*-helix structure. The two peptides possess a significant virucidal activity against herpes simplex virus type 1 (HSV-1) infection *in vitro* [36]. Accordingly, Hp1036 and Hp 1239 had an EC_50_ of 0.43 *μ*M and 0.41 *μ*M, respectively. It has been suggested that these peptides may interact directly with viral membranes by binding to the negatively charged sialic acid units on the virus and inactivate viral particles. Furthermore, these peptides display effective inhibitory proprieties in all stages of HSV-1 life cycle [37]. In fact, they are able to inhibit HSV-1 when it attaches to the cell surface and when it has already bound to the cells but not entered yet and also exhibited potent inhibitory effects against viral particles proliferation in postentry stage. This means that the peptides had the ability to enter the Vero cells and inactivate the mature viral particles [37].

#### 4.4.2. rEv37 Peptide

The rEv37 has the ability to inhibit HSV-1 infection in a concentration-dependent manner at noncytotoxic concentration *via* inhibiting viral entry to the host cells by the low pH-dependent endocytosis pathway [25].

#### 4.4.3. Eval418 and Eval418-FH5 Peptides

The Eval418 is an amphipathic peptide with an *α*-helix structure, identified from *Euscorpiops validus* venom peptide library. It had a dose-dependent inhibitory activity on HSV-1 attachment to host cells. Nevertheless, Eval418 barely inhibited an established viral infection because of low cellular uptake. Thus, a histidine-rich peptide was developed from Eval418 by adding histidine residues to the derivative peptides. The most effective derivative peptide against HSV-1 was Eval418-FH5. This peptide has the same secondary structure as the native peptide but with an enhanced amphiphilicity [38]. Additionally, Eval418-FH5 had a strong viral inhibition, elevated attachment inhibitory activity, and also an improved intracellular uptake and distribution which were manifested by a powerful inhibitory activity against intracellular HSV-1 [38].

### 4.5. Antimeasles Virus (MeV) Peptide

MeV is a single-stranded RNA, negative sense, enveloped virus of the genus *Morbillivirus* within the family of Paramyxoviridae. The virus is enveloped by a lipid membrane studded with many hemagglutinin and fusion proteins. It is the cause of measles, an infection of the respiratory system [39].

#### 4.5.1. Mucroporin-M1 Peptide

Mucroporin-M1 is an artificially scorpion venom-derived peptide that has a highly suppressive ability against MeV with an EC_50_ of 7.15 *μ*g/ml (3.52 *μ*M) and a CC_50_ of 70.46 *μ*g/ml (34.70 *μ*M) [36]. Experimental studies on mucroporin-M1 had shown that MeV deactivation was enhanced by raising the concentration and treatment time by this AVP. Which indicates that mucroporin-M1 mode of action is mediated at least partially by the direct interaction with virus particles surface. Thus, mucroporin-M1 can attach to the virus envelope *via* surface charge interactions and significantly decrease viral infectivity [40].

### 4.6. Anti-Influenza-H5N1 Peptide

The influenza-H5N1 is a subtype of the influenza A virus of the genus *α-influenzavirus* of the family Orthomyxoviridae. H5N1 are roughly spherical viruses with glycoprotein spikes on the surface, its genome consists of eight RNA negative sense fragments that encode 10 proteins [41].

#### 4.6.1. Mucroporin-M1 Peptide

Influenza-H5N1 is another virus inhibited by mucroporin-M1 with an EC_50_ of 2.10 *μ*g/ml (1.03 *μ*M). H5N1 infectivity was suppressed by direct interaction of mucroporin-M1 with viral particles envelope [40].

### 4.7. Antidengue Virus Type 2 (DENV-2) Peptides

Dengue virus type 2 (DENV-2), the cause of dengue fever, is a mosquito-borne, single positive-stranded RNA virus of the family Flaviviridae, genus Flavivirus [42, 43]. DENV-2 is characterized by a relatively smooth surface, with a diameter of approximately 500 Ä, and an electron-dense core surrounded by a lipid bilayer [44].

#### 4.7.1. rEv37 Peptide

The antiviral peptide rEv37 is able to inhibit DEN-2 infection by blocking viral genome release from the endosome into cellular cytoplasm through alkalizing endosomes pH. To explain alkalizing properties of this AVP, authors had postulated that rEv37 may alkalize acidic organelles pH by its own basic properties or could interfere with v-ATPase or other ion channels to modify pH of these organelles. Moreover, rEv37 may suppress DEN-2 infectivity by enlarging the volume of acidic organelles and thus allowing their accumulation [25].

#### 4.7.2. Smp76 Peptide

The Smp76 is an anti-DEN virus peptide with a high inhibitory activity (IC_50_ 10 *μ*g/ml). It prevents DEN virus infection before viral entry by neutralizing extracellular infectious particles [23].

### 4.8. Anti-Zika Virus (ZIKV) Peptide

The Zika virus (ZIKV) is a member of the *Flavivirus* genus of the Flaviviridae family; Zika genome consists of a single-stranded, positive-sense RNA. The virus particles are spherical and small in size, with an envelope protein and membrane proteins. It causes microcephaly and Guillain-Barre syndrome [45].

#### 4.8.1. rEv37 Peptide

As previously mentioned, rEv37 had a great antiviral potential against viruses of the Flaviviridae family, which enter the host cell through clathrin-mediated endocytosis and low pH-dependent fusion. Thus, rEv37 suppresses ZIKV infection in concentration-dependent manner and at noncytotoxic concentration by alkalizing acidic organelles [46].

#### 4.8.2. rSmp76 Peptide

The rSmp76 is an identical peptide to Smp76 but it is recombinantly produced from *Scorpio mauruspalmatus* venom. rSmp76 is able to inactivate established infection effectively with an IC_50_ of 6.63 *μ*mol/l. rSmp76 suppresses the established infection of ZIKV by triggering interferon regulatory transcription factor 3 phosphorylation, leading to the upregulation of IFN-*β* expression and inhibition of viral infection [45, 47].

### 4.9. Anti-SARS-CoV Peptide

Responsible of severe acute respiratory syndrome, SARS-CoV belongs to the Coronaviridae family, genus *Coronavirus*. It is a positive-sense, single-stranded RNA virus. Its genome is composed of 30,000 nucleotides that code for about 10 proteins. It is encapsulated in a small spherical viral particle of about 100 nanometers in diameter [48].

#### 4.9.1. Mucroporin-M1

Mucroporin-M1 had the ability to bind to the viral particles envelope and drastically suppresses SARS-CoV infectivity by direct virucidal action. Thus, the EC_50_ of mucroporin-M1 is of 14.46 *μ*g/ml (7.12 *μ*M) against SARS-CoV [49].

## 5. Key Points on Scorpion Antiviral Peptides

Several scorpion-derived peptides had shown an anti-HCV activity with a wide array of mechanisms of action. Thus, the most effective peptide in terms of the IC_50_ was Smp76 with an IC_50_ of 0.01 *μ*g/ml followed by Ctry2459 and BmKDfsin3 with an IC_50_ of 1.84 *μ*g/ml and of 3.35 *μ*M, respectively. However, considering the variety of mechanisms of action, the most effective peptides were BmKDfsin3 peptide and Hp1090 peptide because of their ability to inhibit the attachment and postentry stages of the viral infection.

Currently, only two scorpion peptides were tested and against HBV; BmKDfsin4 and mucroporin-M1. BmKDfsin4 had the lowest IC_50_ and may inhibit HBV replication. On other hand, mucroporin-M1 had the ability to reduce DNA productions and inhibit intracellular RNA replication.

Five scorpion peptides were tested against herpes simplex virus-1 (HSV-1) so far. The most effective peptide was Hp 1239 and Hp1036 with the lowest EC_50_ and the capacity to inactivate mature viral particles. Other peptides such as rEv37, Eval428, and Eval418-FH5 are effective against HSV-1 but they may just inhibit viral entry.

Considering anti-Zika and antidengue viruses, two peptides had shown an effective antiviral activity; rEv37 and rSmp76 peptide. Had the lowest IC_50_ and the ability to suppress the established infection of ZIKV. While rEv37 suppresses viral infection via alkalizing acidic organelles.

Additional viral infections had been used to assess antiviral activity of scorpion-derived peptides. Thus, Kn2-7 had shown a potent antiviral activity against HIV by damaging virus particle itself. Moreover, mucroporin-M1 was tested against measles, SARS-CoV, and influenza-H5N1 and had shown direct virucidal action and low EC_50_.

## 6. General Features of Antiviral Scorpion Peptides

Commonly, scorpion AVPs are small, cationic, and amphipathic peptides with a net positive charge. In addition to the amphipathicity and charge, hydrophobicity of scorpion AVPs appeared to be essential for these peptides to work as antivirals against enveloped viruses. Several advantages of natural AVPs have been reported, notably their high specificity even in nanomolar range, low toxicity, low molecular weight, and the facility of synthesis. Moreover, they are readily biodegradable by peptidases present in the body, avoiding their possible accumulation in specific organs and reducing the toxic secondary effects [50]. Conversely, a short half-life, immunogenic potential, high production cost, and low oral absorption limit the use of these agents as effective antivirals [51]. Recent advanced research, however, had made it possible to overcome these pharmacodynamics defects through synthesis of amino acid enantiomers, addition of chemical compounds and their nanoparticle formulation [52].

## 7. AVP Improvement Strategies

AVP improvement is aimed at maximizing antiviral activity, potency, ability to cross membrane barriers, and resistance to proteolytic degradation while minimizing toxicity toward the host. Several techniques are described to improve poor ADME (absorption, distribution, metabolism, and elimination) properties of natural antiviral peptides; physiochemical and template-based methods [53]. Accordingly, changes of redundant hydrophobic residues by charged amino acid may increase peptides bioavailability [51]. Other strategies had focused on the enhancement of peptides delivery and permeability by adding cell-penetrating peptides [19]. Peptide delivery is using nanoparticles, prefilled syringes, autoinjectors, and biodegradable microneedles as other alternative techniques to ensure efficient delivery of peptides. AVP stability is increased by their conjugation to polymers for instance polyethylene glycol [19]. Chemical changes in the peptide structure such as shortening peptides, charge, hydrophobicity, and eliminating unstructured regions from helical peptides may improve antiviral activity, decrease cytotoxicity, or provide targeted properties such as electrostatic interactions, membrane spanning, and solubility [54, 55].

## 8. Potential Application of Scorpion Antiviral Peptides against SARS-CoV Viruses

Coronavirus, such as SARS-CoV and MERS-CoV, is highly contagious pathogens with an increased ability to induce lethality in humans with a high potency to cross species barriers and spread across large numbers of populations through human-to-human infection. The threat of the current worldwide COVID-19 pandemic and the enhanced rate of drug-resistant virus strains against existing antiviral drugs necessitates the discovery of new strategies for antiviral therapy. AVPs constitute an excellent alternative as novel therapeutic agents since they have been shown to possess anticoronavirus activity. These peptides may act on several coronaviruses' life cycle stages [5, 20, 56, 57].

The life cycle of coronavirus family generally starts by the fusing of spike protein into the host receptor and entry into the host cell, followed by viral replication and transcription and finally protein production, assembly, and liberation of new virus particles [58]. Thus, according to these viral steps, there are various antiviral mechanisms of action by which AVPs may suppress coronavirus pathogenicity.

### 8.1. Viral Envelope Disruption

It is well-known that positively charged peptides had the ability to interact with lipid rafts rich in sphingolipids and cholesterol negatively charged forming the viral envelope and thus disturb viral membranes [59, 60]. Cationic peptides may also destabilize viral surface proteins enriched in the lipid rafts domains [39]. Various AVPs discovered or derived from scorpion venom peptides have been reported to be positively charged and capable of directly damaging virus particles, such as Kn2-7 that may aggregate and insert into viral envelope and form a pore in viral membrane. Another AVP derived from scorpion venom and has the ability to bind to viral particles and permeabilizing their phospholipid membranes is the Hp1090 [37].

### 8.2. Virus Attachment and Fusion Inhibition

The preliminary attachment of coronaviruses virion to the cellular membrane of the host cell starts by interactions between the viral spike (S) glycoprotein and its receptor [19]. After receptor binding, the virus needs to get into the host cell cytosol. This step is ensured by an acid-dependent proteolytic cleavage of S protein followed by fusion of the viral and cellular membranes within an acidified endosome. There are several scorpion AVPs that may inhibit this first step in the coronavirus's life cycle (e.g., rEv37) which have the ability to suppress viral infection through alkalizing endosomes pH and blocking proteolytic cleavage of S protein and thus inhibiting virus attachment and fusion [57].

### 8.3. Viral Replication Inhibition

Viral replication blockage is another target to curtail coronaviruses infections by interaction and interference with proteins that are essential for viral replication cycle.

Virus infections by interaction and interference with proteins are fundamental for the viral replication cycle. In fact, optimized mucroporin activates the mitogen-activated protein kinases (MAPKs) that lead to decrease in the binding of HNF4*α* to the precore/core promoter region and reduce the expression of HNF4*α* [34].

## 9. Conclusion

Scorpion antiviral peptides are structurally and functionally versatile due to their simple primary structure and could serve as molecular models for the generation of rapid therapeutic candidates for new or emerging outbreaks which pose serious public health threats in the future. Given the unique and promising peptide activity, the potential use of scorpion antiviral peptides in the treatment of virus should be further explored.

## Figures and Tables

**Table 1:** Selected antiviral peptides derived from scorpions and their mode of action against viruses.

Peptide	Source	Characteristics	Targeted virus	Mode of action	IC50	Reference
Ctry2459	*Chaerilus tryznai Chaerilus tricostatus*	Peptides of 13 amino acid residues helical and amphipathic peptide	Hepatitis C virus	Destabilizes the viral structural integrity, thus reducing the initiation of HCV infection	1.84 *μ*g/ml	[27]
Ctry2459-H2 and H3	Derived from Ctry2459	Histidine-rich Ctry2459 peptide	Hepatitis C virus	Reduce the intracellular viral levels	1.08–0.85 *μ*g/ml	[27]
Eval418	*Euscorpiops validus*	12-14 amino acids in length, *α*-helix structures	HSV-1	Inhibition by disruption of initial steps of infection	3.70 *μ*g/ml	[38]
Eval418-FH5	Derived from Eval418	Histidine-rich derivative of Eva1418 peptide	HSV-1	Enhanced inhibition activity with lowest cytotoxicity	0.86 *μ*g/ml	[38]
BmKDfsin4	*Buthus martensii Karsch*	Small cationic peptides composed by 34–51 amino acid residues, which commonly contain six cysteines	Hepatitis B virus	Inhibitory activity against HBV replication	1.26–3.95 *μ*M	[33]
BmKDfsin3	*Buthus martensii Karsch*	38 amino acid residues, which includes six cysteine residues forming three pairs of disulfide bonds	HCV	Inhibit HCV replication and affect the attachment and postentry stages of the viral infection cycle at noncytotoxic concentrations.	3.35 *μ*M	[24]
Mucroporin-M1	*Lychas mucronatus*	Cationic host defense peptide	MeV, influenza-H5N1; SARS-CoV	Virucidal activity	7.15 *μ*g/ml Mev, 14.46 *μ*g/ml SARS-CoV, and 2.10 *μ*g/ml H5N1	[40]
Mucroporin-M1	*Lychas mucronatus*	Cationic host defense peptide	HBV	Inhibition of viral replication by decreasing expression of important HBV replication factors	11 *μ*M	[34]
Hp1036	*Heterometrus petersii*	Amphipathic *α*-helical peptide	HSV-1	Inhibition of cell entry by blocking viral-host membrane fusion	0.43 *μ*M	[26]
Hp1239	*Heterometrus petersii*	Amphipathic *α*-helical peptide	HSV-1	Inhibition of cell entry by blocking viral-host membrane fusion	0.41 *μ*M	[26]
Hp1090	*Heterometrus petersii*	Amphipathic *α*-helical peptide	HCV	Inhibition of viral replication	7.62 *μ*g/ml (5.0 *μ*M),	[63]
kn2-7	Developed from BmKn2 peptide (*Mesobuthus martensii*)	13 amino acid residues, basic, alpha-helical peptide	HIV-1	Inhibition by direct interaction with viral particle	2.76 *μ*g/ml (1.65 *μ*M)	[22]
rEv37	Derived from Ev37 isolated form *Euscorpiops Validus*	78 amino acid residues and is a scorpine-like peptide, including a CS*α*/*β* motif. N terminus is likely an *α*-helix, and C terminus is a conserved CS*αβ* scaffold	(DENV-2), (HCV), (ZIKV), and (HSV-1)	Inhibit DENV-2 infection and suppress HCV and ZIKV infections	10 *μΜ*	[23]
Smp76	*Scorpio maurus palmatus*	76 amino acids with six residues of cysteine. Cysteine-stabilized *α*/*β* fold, and three disulfide bridges	DENV, HCV, and ZIKV	Inhibits the ability of HCV virus to infect the host cells	0.01 *μ*g/ml	[47]
